# Effectiveness of Antalgic Therapies in Patients with Vertebral Bone Metastasis: A Protocol for a Systematic Review and Meta-Analysis

**DOI:** 10.3390/ijerph18083991

**Published:** 2021-04-10

**Authors:** Antonio Jose Martin-Perez, María Fernández-González, Paula Postigo-Martin, Marc Sampedro Pilegaard, Carolina Fernández-Lao, Eduardo Castro-Martín, Lydia Martín-Martín, Mario Lozano-Lozano

**Affiliations:** 1 Department of Internal Medicine, San Cecilio University Hospital, Hospital Universitario San Cecilio, 18016 Granada, Spain; ajmartinperez@correo.ugr.es; 2 Clinical Medicine and Public Health Doctoral Studies, University of Granada, 18010 Granada, Spain; 3 Department of Physical Therapy, Faculty of Health Sciences, University of Granada, 18010 Granada, Spain; mariafg@correo.ugr.es; 4 Biohealth Research Institute in Granada (ibs.GRANADA), 18016 Granada, Spain; paulapostigo@ugr.es (P.P.-M.); carolinafl@ugr.es (C.F.-L.); eduardoc@ugr.es (E.C.-M.); mlozano@ugr.es (M.L.-L.); 5 Sport and Health University Research Institute (iMUDS), “Cuídate” Support Unit for Oncology Patients (UAPO-Cuíate), 18010 Granada, Spain; 6 Occupational Science and Occupational Therapy, The Research Unit for User Perspectives and Community-Based Interventions, Department of Public Health, University of Southern Denmark, 5230 Odense, Denmark; mpilegaard@health.sdu.dk; 7 REHPA, The Danish Knowledge Centre for Rehabilitation and Palliative Care, Odense University Hospital, 5000 Odense, Denmark

**Keywords:** systematic review, neoplasm metastasis, bone and bones, spine, pain, protocol

## Abstract

There is no systematic review that has identified existing studies evaluating the pharmacological and non-pharmacological intervention for pain management in patients with bone metastasis. To fill this gap in the literature, this systematic review with meta-analysis aims to evaluate the effectiveness of different antalgic therapies (pharmacological and non-pharmacological) in the improvement of pain of these patients. To this end, this protocol has been written according to the Preferred Reporting Items for Systematic review and Meta-Analysis Protocols (PRISMA-P) and registered in PROSPERO (CRD42020135762). A systematic search will be carried out in four international databases: Medline (Via PubMed), Web of Science, Cochrane Library and SCOPUS, to select the randomized controlled clinical trials. The Risk of Bias Tool developed by Cochrane will be used to assess the risk of bias and the quality of the identified studies. A narrative synthesis will be used to describe and compare the studies, and after the data extraction, random effects model and a subgroup analyses will be performed according to the type of intervention, if possible. This protocol aims to generate a systematic review that compiles and synthesizes the best and most recent evidence on the treatment of pain derived from vertebral metastasis.

## 1. Introduction

Cancer is one of the most common diseases worldwide and further one of the most disturbing. Half of cancer patients suffer from bone metastasis. This is the third-most frequent site for distant metastasis after lung and liver [1]. The spine is the most common place for skeletal metastasis because of its rich blood supply and sinusoidal vascular distribution [2]. Between 40–70% of bone metastasis have serious consequences, such as vertebral lesions and spinal cord compression, leading to neurological deficits and pain [3,4].

In total, 66.4% of cancer patients with metastasis suffer from pain, and it is a prevalent symptom during the whole process [5]. In particular, pain is one of the symptoms that causes the greatest limitation in functional activity, making its management a challenge not only for professionals but also for patients [6]. The character of the pain in bone metastasis can be somatic (musculoskeletal), neuropathic (caused by nerve irritation) or mixed, which is the most common [7]. Pain from bone metastasis can be incidental or spontaneous; incidental pain is usually associated with movement, so achieving normal pain-free movement becomes very difficult for these patients [6]. Severe pain occurs during everyday activities such as walking, sitting, coughing or changing posture [8]. This persistent pain is devastating for the quality of life (QoL) of those suffering from cancer [9], and it is associated with a cluster of cumulative consequences such as social isolation, emotional and spiritual distress and fear of death with suffering [10]. Collectively, this shows that pain is one of the main cancer problems and one of the most disabling.

The treatment of metastasis is complex, being highly variable depending on the patient’s prognosis and the specific type of lesion (blastic or lytic). Chemotherapy and radiation therapy are the most efficient types of cancer treatments, but they are not the only therapeutic options. The management of bone metastasis entails a multidisciplinary approach integrating various disciplines such as surgery, radiation oncology and medical oncology [11]. One of the most important objectives in bone metastasis treatment is to maximize pain control. Conventional external radiotherapy is usually a very common option in almost 60% of the cases for the treatment of this type of lesion, with the aim of alleviating pain, an objective that is fully achieved in 25% of cases. Surgical treatment and interventional radiology are therapeutic options reserved for those lesions with a more compromised approach [12]. As 50% of bone metastasis are considered osteolytic lesions, antiresorptive treatment with bisphosphonates or denosumab plays an important role [13]. However, current literature on cancer treatment has so far not only focused on patients with vertebral bone metastasis and the effectiveness on pain. Turkiewicz and Reale conducted a narrative review in 2001 on medical treatment for pain management in bone metastases [14]. Jehn et al. [11] proposed an algorithm for the treatment of bone metastasis combining treatments such as surgical treatment, osteoprotective therapy, pain therapy or radiotherapy. However, they do not focus on the effectiveness of pain management treatments. Another recent review by Ahmad et al. [15] investigated different forms of pain management, but again, their population was not restricted to only include patients with vertebral bone metastasis.

In addition to pharmacological treatment, some non-pharmacological interventions seem to be promising as resources for the management of pain in these patients. Physical recovery is an alternative for pain control and improvement of spinal instability in cases where surgical options are contraindicated, rejected by the patient or are of questionable cost-effectiveness [12]. Since bone metastasis often cause pain [16,17], this contributes to the physical deconditioning of the patient, induced by movement avoidance behavior. This may also maintain the pain experience of the patients and leads to a circle that is difficult to break [18]. Loss of physical activity predisposes the patient to secondary complications in the form of bone fractures [19], high levels of cancer-induced fatigue [20] or increases in body fat [21], which again increases the patient’s physical vulnerability at this stage of the disease. Some reviews have already examined the combination of pharmacological and non-pharmacological treatment for bone metastasis, such as those proposed by Buga et al. [7] and by Mercadante et al. [8], who include different treatments to control pain; nevertheless, they do not focus on vertebral bone metastasis, and furthermore, they are not up to date. Regarding non-pharmacological treatments, we found studies that have investigated the effectiveness of a full-body massage on pain intensity [22] and the need of doing exercise [7]; additionally, attempts have been made to use hypnosis in order to control pain [23].

There are a large number of publications that have addressed pharmacological treatment in bone metastasis, but there is not yet a systematic review that has synthesized scientific evidence about this type of treatment and its effectiveness in patients with vertebral bone metastasis. Likewise, this is also the case for non-pharmacological treatment. Hence, it is necessary to carry out a systematic review of the literature that combines the knowledge of the different treatments in vertebral bone metastasis. Therefore, the aim of the systematic review is to evaluate the effectiveness of different antalgic therapies (pharmacological and non-pharmacological) in the improvement of pain in patients with vertebral bone metastasis.

## 2. Materials and Methods

The present protocol has been registered in PROSPERO (Prospective International Register of Systematic Reviews; registration number CRD42020135762). This protocol has been written according to the Preferred Reporting Items for Systematic review and Meta-Analysis Protocols (PRISMA-P) [24]. The studies that will be included in the systematic review will be evaluated according to criteria established in this protocol.

### 2.1. Eligibility

In this systematic review, we will include published articles with cancer patients who have painful vertebral bone metastasis in any location, from any type of cancer and any type of metastasis.

The inclusion criteria for the studies will be: (a) having a methodological design of clinical trials; (b) on adults (18+ years) with vertebral bone metastasis suffering from pain; (c) with patients receiving any pharmacological or non-pharmacological intervention; (d) that assessed pain as an outcome measure; (e) written in English or Spanish; (f) full-text articles, otherwise the authors will be contacted.

Exclusion criteria will be: (a) reviews, monographs, guidelines, surveys, commentaries, editorials, case reports, conference papers, unpublished data and letters; and (b) animal or in vitro studies.

### 2.2. Outcomes

The outcome will be obtained from pain scores from baseline to the last available follow-up. Pain needs to be measured using any validated assessment instrument.

### 2.3. Information Sources

We will perform comprehensive systematic searches within the following electronic databases to identify all relevant studies published from date of inception: (a) MEDLINE (via PubMed); (b) Scopus (c) Web of Science; (d) The Cochrane Library. These four academic and professional databases have been chosen in consultation with an experienced medical subject librarian as the most appropriate for this review.

### 2.4. Search Strategy

The search strategy for this study will include MeSH terms and free entry terms (in English), as appropriate. The terms will be combined with Boolean operators (AND, OR) and search techniques such as truncation, phrases marks or wildcard will be used with slight modifications in each database. The terms of each category have been obtained by consulting other previous reviews [25,26,27] and in consultation with the experienced medical subject librarian mentioned above. MeSH terms such as “pain [MeSH Terms],” “neoplasm [MeSH Terms]” or “spine [MeSH Terms]” will be used. Additionally, entry terms such as “tumor*[tiab]” or “neoplasm*[tiab].”

### 2.5. Selection Process

The selection process for the included studies will be carried out in three phases and developed by two external researchers. In case of discrepancies, a third researcher will be included, and a consensus will be reached. Firstly, EndNote X9 bibliographic management software will be used to compile the bibliographic references for each of the included databases. The systematic review software Rayyan QCRI, both its Web version and its iPad version, will be used to carry out the elimination of duplicates, as well as the screening and selection of studies to be included in this systematic review. This software has been widely used in research [28,29]. Secondly, once duplicates have been removed, the titles and abstracts of all identified studies by the search strategy will be examined, and irrelevant studies will be excluded. Thirdly, the full texts of studies to be included in the second screening phase will be obtained and the eligibility criteria will be assessed. Any disagreement about the eligibility of particular studies will be resolved by discussion with an external reviewer.

In the same way, the reference lists of identified and included studies will be examined (advanced search), in order to identify any study not filtered by the search equation. Just before the qualitative and quantitative analysis of the results, and writing the paper, the search will be performed again using the same equation, to retrieve possible additional studies and analyze their possible inclusion. In addition, search alerts will be set up.

### 2.6. Extraction and Qualitative Synthesis of the Data

Data extraction will be performed for the study quality assessment. A spreadsheet specifically designed to facilitate the process of selection, data extraction and analysis will be used, including: (1) first author, (2) year of publication, (3) clinical entity, (4) sample size, (5) intervention used (pharmacological: drug, dose, prescription...) and non-pharmacological (type of intervention, dose, temporality...), (6) details of the control or comparative group, (7) outcomes, (8) adverse effects and (9) main results.

A narrative synthesis will be used to describe and compare the studies (quantitative synthesis regarding measures of central tendency, risk coefficient, 95% confidence intervals and the level of statistical significance of the *p*-value, as appropriate). There will be no minimum number of studies required for synthesis. The search and inclusion/exclusion methods will be presented in a flow chart [30]. Considering the inconsistency of the assessment tools, data consolidation will be used to describe the main results. The main quality variables and study results will be summarized and tabulated. A comprehensive descriptive account of the adverse effects will be reported.

Finally, future recommendations for research, trials and reporting will be made based on the findings of this review.

### 2.7. Extraction and Quantitative Synthesis of the Data

After the data extraction, the reviewers will determined if there is a possibility of performing a meta-analysis by considering if the heterogeneity is moderate or strong as assessed by I2 (less than 25% no heterogeneity, 25–49% low heterogeneity, 50–74% moderate heterogeneity and 75% or greater high heterogeneity) [31]. The random effects model will be used for the analysis. Means and Standard Deviations (SD) of measures will be used to compare the effect size of improvement in pain management in patients with vertebral bone metastasis. We will also perform subgroup analyses, if possible, according to the type of intervention.

### 2.8. Risk of Bias: Quality Assessment

The methodological quality of the included studies will be examined independently by two reviewers using the Cochrane Collaboration’s Risk of Bias tool, resolving disagreements by consensus, with the third reviewer being contacted if a consensus is not reached. We will assess the risk of bias potentially, including random allocation, concealment of allocation, blind subjects, blind therapists, blinded outcome assessment, selective outcome reporting, and incomplete outcome data from Cochrane Collaboration’s Risk of Bias tool. The resulting findings will be summarized, and notes will be taken of the papers that passed screening for inclusion in the narrative review but did not meet standards for determining efficacy based on the reported data.

## 3. Expected Results

This article presents the protocol for a systematic review of the effectiveness of different pharmacological and non-pharmacological treatments on pain relief among patients with vertebral bone metastasis. The decrease in the number of skeletal related events (SRE; pathologic fracture, spinal cord compression, necessity for radiation to bone or surgery to bone) is usually the main variable in studies on the treatment of vertebral metastasis [32]. However, we will focus on pain relief as the primary endpoint because of its high prevalence and impact on patients’ QoL [5,9]. Previously published systematic reviews do not focus on this particular type of metastasis, do not address analgesic treatment or restrict the results to specific types of cancer [11,15,32]. Our systematic review will be the first of its kind to investigate the benefit of analgesic treatment in patients with vertebral bone metastasis.

This protocol aims to generate a systematic review that compiles and synthesizes the best and most recent evidence on the treatment of pain derived from vertebral metastasis. We aim to disseminate the scientific evidence on this subject and, more specifically, to distinguish on the basis of the published evidence whether pharmacological or non-pharmacological treatments are effective, which doses or radiotherapy regimens have achieved the best results, which drugs are the most recommendable due to their greater effectiveness and fewer side effects, which measures achieve earlier pain relief, and which are more long-lasting remedies.

Despite these ambitions, it is likely that we will encounter some limitations, derived above all from the heterogeneity of the different studies to be included, which may hinder the quantitative analysis of the data, as well as the drawing of conclusions that can be extrapolated to all patients. However, we hope that these limitations will not bias the results and sensitivity analysis methods will be used to ensure the quality of the conclusions. In addition, the language inclusion criteria include only English and Spanish. In terms of strengths, only randomized controlled clinical trials, studies with the most scientific evidence, will be included. In addition, to our knowledge, there is no systematic review that fills these gaps in the literature, making such a broad, comprehensive, and rigorous approach to find interventions specifically aimed at bone pain due to vertebral metastasis.

## 4. Conclusions

In this manuscript, a detailed description of the systematic review protocol with a meta-analysis of pharmacological and non-pharmacological interventions for the management of pain from vertebral bone metastasis is presented. The described methodology of the review, including the search of the recommended databases, the identification and screening of records, the data extraction, the methods used to assess the quality of the included studies, and the qualitative and quantitative analysis of the studies, has been described in detail. Undertaking such a systematic review is challenging and, despite the potential limitations, the future quality of the work is assured by the fact that the study methodology has been registered in PROSPERO and that the results will be published after a peer review process.

## Data Availability

Not applicable.
